# Correlation of Gastroesophageal reflux disease Assessment Symptom Questionnaire to impedance-pH measurements in children

**DOI:** 10.1177/2050312117745221

**Published:** 2017-12-12

**Authors:** Sittichoke Prachuapthunyachart, Chaowapong Jarasvaraparn, David A Gremse

**Affiliations:** 1 Division of Pediatric Gastroenterology, Hepatology and Nutrition, University of Nebraska Medical Center, Omaha, NE, USA; 2 Department of Pediatrics, University of South Alabama, Mobile, AL, USA; 3 Division of Pediatric Gastroenterology, Hepatology and Nutrition, University of South Alabama, Mobile, AL, USA

**Keywords:** Gastroesophageal reflux disease, multichannel intraluminal impedance-pH study, Gastroesophageal reflux disease Assessment Symptom Questionnaire, reflux index, impedance score, vomiting, choking, abdominal pain

## Abstract

**Background:**

Esophageal multichannel intraluminal impedance-pH monitoring has become one of the preferred tests to correlate observed reflux-like behaviors with esophageal reflux events. The Gastroesophageal reflux disease Assessment Symptom Questionnaire is a validated tool used to distinguish infants with gastroesophageal reflux disease from healthy children. The aim of this study was to determine whether the Gastroesophageal reflux disease Assessment Symptom Questionnaire composite symptom scores and individual symptom scores correlate with outcomes in esophageal multichannel intraluminal impedance-pH monitoring.

**Methods:**

A total of 26 patients with gastroesophageal reflux disease–associated symptoms, aged 0–2 years, for whom both esophageal multichannel intraluminal impedance-pH monitoring and Gastroesophageal reflux disease Assessment Symptom Questionnaire survey results were available were included in the study. Gastroesophageal reflux disease Assessment Symptom Questionnaire score data were collected from a 7-day recall of parent’s responses about the frequency and severity of gastroesophageal reflux disease symptoms, which determined the individual symptom scores. The composite symptom scores is the sum of all individual symptom scores. Multichannel intraluminal impedance-pH study results were compared to Gastroesophageal reflux disease Assessment Symptom Questionnaire data using Pearson correlation.

**Results:**

Among 26 patients, a total number of 2817 (1700 acid and 1117 non-acid) reflux episodes and 845 clinical reflux behaviors were recorded. There were significant correlations between the reflux index and the individual symptom scores for coughing/gagging/choking (r^2^ = 0.2842, p = 0.005), the impedance score and individual symptom scores for coughing/gagging/choking (r^2^ = 0.2482, p = 0.009), the reflux symptom index for acid reflux-related coughing/gagging/choking and the individual symptom scores for coughing/gagging/choking (r^2^ = 0.1900, p = 0.026), the impedance score and individual symptom scores for vomiting (r^2^ = 0.1569, p = 0.045), and the impedance score and the composite symptom scores (r^2^ = 0.2916, p = 0.004). However, there were no significant correlations between fussiness, irritability, or abdominal pain–related multichannel intraluminal impedance-pH results and the individual symptom scores for abdominal pain.

**Conclusion:**

The impedance scores from multichannel intraluminal impedance-pH studies correlate with coughing/gagging/choking and vomiting in infants with gastroesophageal reflux disease. There are no significant correlations among the reflux index and impedance score versus the Gastroesophageal reflux disease Assessment Symptom Questionnaire scores for abdominal pain. We conclude that in infants with gastroesophageal reflux disease, multichannel intraluminal impedance-pH studies are more likely to demonstrate an association between gastroesophageal reflux disease and symptoms of coughing, gagging, or choking compared to an association between gastroesophageal reflux disease and pain in infants.

## Introduction

Gastroesophageal reflux (GER) is the physiologic passage of gastric contents into the esophagus that commonly occurs in healthy infants. In contrast, gastroesophageal reflux disease (GERD) is present when esophageal reflux causes troublesome symptoms and/or complications.^1^ The prevalence of GERD in preterm infants ranges between 1.8% and 8.2%.^2^ The limitation of esophageal pH monitoring for evaluation of GERD in infants is that it only records acid reflux events. This limitation decreases the detection of total esophageal reflux events in the postprandial period when stomach contents may be non-acidic for up to 2 hours after a meal. Therefore, for infants who are fed every 2–3 hours, the pH study might significantly underestimate the amount of esophageal reflux.^3^ Therefore, esophageal multichannel intraluminal impedance-pH (MII-pH) monitoring has been increasingly employed for the evaluation of esophageal reflux in pediatric and adult patients. MII-pH monitoring detects liquid, gas, or mixed esophageal reflux in addition to acid or non-acid reflux.^4^ Esophageal MII-pH monitoring significantly increases the detection of esophageal reflux events in infants compared to conventional esophageal pH monitoring.^5^

The diagnosis of GERD in infants is suspected on the basis of symptoms; however, invasive testing is often used in the diagnostic evaluation. In order to avoid invasive tests such as esophageal MII-pH monitoring or endoscopy with biopsy, non-invasive diagnostic tools have been sought. One such non-invasive instrument is the validated, structured questionnaire of clinical observations for GERD, the Gastroesophageal reflux disease Assessment Symptom Questionnaire (GASQ).^6,7^

The objective of our study was to determine whether the GASQ composite symptom scores (CSS) and individual symptom scores (ISS) correlate with outcomes in esophageal MII-pH monitoring.

## Methods

### Study design

This study is a retrospective cross-sectional questionnaire survey designed to evaluate for a correlation between a GASQ scores and MII-pH parameters in infants. Consecutive patients aged less than 24 months old at the University of South Alabama Children’s and Women’s Hospital who were referred for evaluation of suspected GERD between October 2014 and September 2015 were eligible for inclusion in the study. Patients with persistent vomiting, coughing/gagging/choking, or abdominal pain had suspected GERD. Invasive testing was performed as per the GERD practice guidelines. For example, diagnostic evaluation for GERD is warranted in infants with GERD symptoms combined with poor feeding or failure to thrive. The Institutional Review Board of the University of South Alabama approved the study.

The medical records of 29 patients who underwent esophageal MII-pH monitoring and had complete GASQ questionnaires were reviewed. Patients with prior history of gastrointestinal surgery and other gastrointestinal conditions that may mimic the symptoms of GERD, that is, eosinophilic esophagitis, esophageal motility disorders, congenital esophageal abnormalities (e.g. tracheoesophageal fistula), and cardiopulmonary diseases, were excluded. Three patients with incomplete GASQ and/or MII-pH results were excluded, yielding 26 patients who were included in the analysis.

### Questionnaire

The GASQ^7^ assesses the frequency and usual severity of symptoms based on 7-day recall. It consists of parental reporting on six items (vomiting/regurgitation, coughing/gagging/choking, abdominal pain, burping/belching, difficulty swallowing, refusal to eat), identifying the number of episodes during the past week and the severity of each symptom based on a Likert scale from 1 (not at all severe) to 7 (most severe). Parents or guardians who lived in the same household as the infants completed the questionnaires.

### Questionnaire scoring

An individual score of each symptom (ISS) and a composite symptom score (CSS) were calculated based on the GASQ questionnaire results. The ISS was calculated by multiplying the total number of events for each symptom reported over the past 7 days by the severity of each symptom reflected in the Likert scale result reported by the parent or caregiver. The CSS was calculated as the sum of the ISSs.^7^

### MII-pH study data

Esophageal impedance-pH catheters with a 2.13-mm diameter containing seven impedance sensors (ComforTEC, Sandhill Scientific, Inc., Highlands Ranch, CO) were used for the study. MII-pH data were collected utilizing a ZepHr recorder (Sandhill Scientific) and analyzed with BioView version 1.2 software (Sandhill Scientific). The insertion depth of the catheter was estimated by calculating 87% of the distance from the insertion site to the gastroesophageal junction using the formula by Strobel et al.^8^ The final position of MII-pH catheter was confirmed by chest X-ray between the 7th and 9th thoracic vertebral bodies. Parents/caregivers were instructed on how to record symptoms whether the study was performed in the inpatient or outpatient setting. Proton pump inhibitors (PPIs) were discontinued for 7 days and histamine 2-receptor antagonists (H2RAs) were stopped at least 48 h prior to esophageal MII-pH monitoring. Any patients who were receiving continuous gastric feeding were converted to intermittent bolus feeding during esophageal MII-pH monitoring. Thus, all patients in this study received oral or intermittent bolus tube feeding during the esophageal MII-pH monitoring. After completion of the study, the data were reviewed and analyzed by the pediatric gastroenterologist (D.A.G.). Esophageal reflux events were defined by a retrograde change in impedance >50% from baseline for at least two distal channels. Reflux events were classified as acid (pH < 4) or non-acid based on simultaneous pH monitoring. A symptom-reflux association analysis from the data was performed to assess for a temporal relation between parent-reported symptoms and esophageal reflux events. The data collected for analysis included the following:

- Symptom correlation to reflux = the numbers of reflux episode that correlated to symptom (vomiting, coughing/gagging/choking, and abdominal pain);
- Reflux index (RI) = the percentage of time that the esophageal pH was <4 during the study (total esophageal acid exposure);
- Impedance score (IS) = total number of acid and non-acid esophageal reflux events;
- Reflux symptom index (RSI) = the percentage of symptoms related to esophageal reflux episodes;
- Reflux symptom sensitivity index (RSSI) = the percentage of reflux episodes associated with symptoms.

These indices were considered abnormal when RI >10%, RSI ≥50%, and RSSI ≥10%.^9^

### Statistical analysis

A single researcher (S.P.) collected data on demographics, GASQ results, and MII-pH study results. The results are presented as percent, mean, and standard deviation. The RI, IS, RSI, and RSSI were compared with GASQ results (ISS and CSS score) using analysis of variance (ANOVA) and Pearson correlation. Statistical significance was defined as a p value of less than 0.05.

## Results

### Patient characteristics and GASQ score

A total of 26 infants were included in our study for whom complete GASQ survey scoring and esophageal MII-pH monitoring data were available. The demographics and GASQ scores are shown in Table 1. The mean age of our study subjects was 0.31 ± 0.35 years (113 days). The majority of study subjects were male (57.7%) and Caucasian (76.9%, African-American 23.1%). The ISS values for the most commonly reported symptoms within the GASQ score were vomiting/regurgitation (3657/8189; 45%), abdominal pain (1772/8189; 22%), and coughing/gagging/choking (1666/8189; 20%). The CSS values were ≥27 in 23/26 study subjects (88.5%). Dietary history indicated that study subjects received the following: Neocate (10/26; 38.5%), Elecare (10/26; 38.5%), and one each (3.8%) of Puramino, Alimentum, Enfamil AR, Gentlease, breast milk, and breast milk supplemented with Similac Advance. There was no significant difference in the reporting of symptoms (ISS and CSS) for subjects who received amino acid–based formulas. The median of CSSs for Neocate and Elecare was 344 (interquartile range (IQR): 108.5–481) and 381 (IQR: 97–495), respectively.

**Table 1.** Demographics and GASQ score.

Variable	Patients population
Age (years, mean ± SD)	0.31 ± 0.35
Gender (Male/Female)	15 (57.7%)/11 (42.3%)
Race (Caucasian/African American)	20 (76.9%)/6 (23.1%)
ISS of vomiting/regurgitation (total of all patients)	3657 (45%)
ISS of coughing/gagging/choking (total of all patients)	1666 (20%)
ISS of abdominal pain (total of all patients)	1772 (22%)
ISS of burping/belching (total of all patients)	562 (6%)
ISS of difficulty swallowing (total of all patients)	436 (5%)
ISS of refusal to eat (total of all patients)	96 (1%)
CSS (range)	8–679

GASQ: Gastroesophageal reflux disease Assessment Symptom Questionnaire; SD: standard deviation; ISS: individual symptom scores; CSS: composite symptom scores.

### Analysis of MII-pH data

A total number of 2817 (1700 acid; 60% and 1117 non-acid; 40%) esophageal reflux episodes and 845 clinical reflux behaviors (vomiting 113; 13%, coughing/gagging/choking 224; 26% and abdominal pain/fussiness 508; 60%) were recorded. The RI was >10% in 8 of 26 study subjects (30%). Additionally, the RSI was ≥50% in 16 of 26 study subjects (61.5%), and the RSSI was ≥10% in 3 subjects (11.5%). A summary of reflux associated symptoms, the RSI, and the RSSI is shown in Table 2.

**Table 2.** The summary of symptom correlated to reflux, reflux symptom index (RSI), and reflux symptom sensitivity index (RSSI) from MII-pH study.

Parameters	All reflux		
	Vomiting	Coughing/gagging/choking	Abdominal pain/fussiness
Symptom correlation to reflux (acid/non-acid)	79 (42/39)	115 (52/68)	190 (129/67)
Reflux symptom index (%), median (IQR)	72.5 (0–100)	39 (0–72)	36 (21.25–50.75)
Reflux symptom sensitivity index (%), median (IQR)	2 (0–4.25)	6 (0–12)	6.5 (2–20.5)

IQR: interquartile range; MII-pH: multichannel intraluminal impedance-pH.

### Correlation between GASQ and MII-pH data

The correlation between the severity of GASQ symptoms (vomiting/regurgitation, coughing/gagging/choking when eating, abdominal pain, burping/belching, difficulty swallowing, and refusal to eat) and the parameters of the MII-pH study results (RI, IS, RSI, and RSSI,) was assessed (Tables 3 and 4). Analysis for associations between the GASQ ISS and the MII-pH parameters yielded only one significant correlation between the ISS for vomiting/regurgitation and IS (R^2^ = 0.1569, p = 0.0452). No significant correlation was found between the RI, RSI, and RSSI and the CSS. Additionally, the GASQ individual symptom score for coughing/gagging/choking was significantly associated with the RI (R^2^ = 0.2842, p = 0.0050), IS (R^2^ = 0.2483, p = 0.0096), and the RSI for acid reflux-related coughing/gagging/choking (R^2^ = 0.1900, p = 0.0260). Moreover, only the CSS was significantly correlated with IS from the MII-pH parameters (R^2^ = 0.2916, p = 0.0044). In contrast, there was no significant correlation between the ISS for abdominal pain, burping/belching, difficulty swallowing, or refusal to eat with any of the MII-pH parameters in our study. Moreover, the occurrences of vomiting, coughing/gagging/choking, or abdominal pain recorded by parents during the MII-pH study were not significantly related to the CSS.

**Table 3.** Relationship between ISS and CSS from GASQ and RI, IS from MII-pH study (R^2^, p value).

MII-pH parameters	Vomiting/regurgitation ISS	Coughing/gagging/choking ISS	Abdominal pain ISS	CSS
RI	0.0315	0.2842	0.1199	0.1262
	(p = 0.3861)	(p = 0.0050)	(p = 0.0832)	(p = 0.0749)
IS	0.1569	0.2483	0.0891	0.2916
	(p = 0.0452)	(p = 0.0096)	(p = 0.1385)	(p = 0.0044)

GASQ: Gastroesophageal reflux disease Assessment Symptom Questionnaire; ISS: individual symptom scores; CSS: composite symptom scores; RI: reflux index; IS: impedance score; MII-pH: multichannel intraluminal impedance-pH.

**Table 4.** Relationship between ISS of vomiting, coughing/gagging/choking, and abdominal pain from GASQ and RSI, RSSI from MII-pH study (R^2^, p value).

MII-pH parameters	ISS
RSI acid-related vomiting	0.1445 (p = 0.0908)
RSI non-acid-related vomiting	0.0010 (p = 0.8771)
RSSI acid-related vomiting	0.1192 (p = 0.0840)
RSSI non-acid-related vomiting	0.0265 (p = 0.4267)
RSI acid-related coughing/gagging/choking	0.1900 (p = 0.0260)
RSI non-acid-related coughing/gagging/choking	0.0420 (p = 0.3513)
RSSI acid-related coughing/gagging/choking	0.0094 (p = 0.6383)
RSSI non-acid-related coughing/gagging/choking	0.0002 (p = 0.94696)
RSI acid-related abdominal pain	0.0320 (p = 0.3818)
RSI non-acid-related abdominal pain	0.0012 (p = 0.8690)
RSSI acid-related abdominal pain	0.0086 (p = 0.6526)
RSSI non-acid-related abdominal pain	0.0096 (p = 0.6334)

ISS, individual symptom scores; RSI: reflux symptom index; RSSI: reflux symptom sensitivity index; GASQ: Gastroesophageal reflux disease Assessment Symptom Questionnaire; MII-pH: multichannel intraluminal impedance-pH.

## Discussion

Parent-reported questionnaires based on caregiver observed behaviors have been developed to identify infants with symptoms of GERD.^6,7^ Symptoms in infants are considerably different than GERD symptoms manifested by older children and adults.^6^ GERD symptoms in infants are most frequently assessed in these questionnaires by eliciting responses regarding a variety of infant specific behaviors including regurgitation, crying around feeding, refusal of feeding, back arching, and difficulty breathing. Survey instruments that measure pediatric GERD symptoms include the GERD Assessment Symptom Questionnaire (GASQ), the Infant GERD questionnaire (I-GERQ), and the Infant GERD questionnaire Revised (I-GERQ-R). The I-GERQ standardizes history taking in parents utilizing 138 items with 23 items added during the course of the first assessment. This questionnaire is a reliable and clinically responsive measure of infant GERD,^10,11^ but its length limits its routine use in clinical practice. Therefore, the I-GERQ-R was created by decreasing the I-GERQ survey to 12 items based on parental feedback.^12^ The GERD Assessment Symptom Questionnaire (GASQ) was also developed to assess for GERD symptoms.^7^ Validation of the GASQ survey was conducted using a single visit parallel design in four clinical centers. The mean CSS and ISS were higher in infants (p < 0.001 and p < 0.05, respectively) with GERD than in those without GERD, indicating that this questionnaire can differentiate between infants with GERD versus healthy infants. The GASQ has not been compared with objective diagnostic tests for GERD such as endoscopy, esophageal pH-metry, or esophageal MII-pH monitoring. This study is the first to compare GASQ survey responses with esophageal MII-pH study results.

Herein we reported the comparison between GASQ and esophageal MII-pH study parameters. Acid reflux episodes occurred more often than non-acid reflux episodes in our study (60% acid and 40% non-acid). There is no significant difference between acid and non-acid reflux in our study. Interestingly, some studies showed non-acid reflux episodes were more common than acid reflux.^13–15^ In contrast, some studies demonstrated weakly acid reflux events were more dominant than acid reflux events.^9,16–18^ Since our patients discontinued acid suppression therapy with PPIs or H2RAs for at least 48 h prior to the procedure, this may explain why the acid reflux episodes were more common.

Only the symptoms of vomiting/regurgitation and coughing/gagging/choking correlated with esophageal reflux events recorded during esophageal MII-pH monitoring in this study. In contrast, there was no significant correlation between the abdominal pain, burping/belching, difficulty swallowing, refusal to eat, and the MII-pH study parameters. This observation correlates with conclusions from placebo-controlled trials of infants with GERD symptoms that found no significant improvement in irritability in infants treated with PPIs compared to placebo. Moore et al.^19^ found a poor correlation between symptoms of irritability and esophageal acid exposure during omeprazole treatment in infants when no significant decrease in crying occurred after a reduction in the RI was observed in the treated infants. However, the results of this study support the observations reported by Funderburk et al.^9^ who concluded that only gagging significantly correlated with esophageal reflux events in their patient population.

Funderburk et al.^9^ reported the value of the RSI and the symptom association probability (SAP) in interpreting the results of esophageal MII-pH monitoring in infants. Conversely, Lüthold et al.^15^ showed a poor inter-parameter association among the RSI, RSSI, and SAP that resulted in disagreements in the diagnostic classification of GERD in study subjects. Another study demonstrated a better correlation between MII-pH study results and reflux symptoms in infants (<12 months old) compared to older children.^5^

Orenstein et al. validated the I-GERQ survey instrument in a study comparing 100 normal infants and 35 infants diagnosed with GERD by pH-metry or esophageal biopsy. Although the control population had a high prevalence of reflux symptoms such as regurgitation (40%), respiratory symptoms, crying (17%), arching (10%), or hiccups (36%), many symptoms were significantly more prevalent in the GERD group than in healthy controls.^11^ Conversely, Salvatore et al. evaluated the predictive value of I-GERQ in comparison with esophageal pH monitoring, histology, and clinical score and found that the I-GERQ cut-off score was positive in 81% infants with normal biopsy and pH study results yet failed to identify 26% infants with GERD. They concluded that I-GERQ was poorly predictive for the severity of GERD.^20^ Størdal et al. compared the results of a 7-point questionnaire and esophageal pH study results in 7- to 16-year-old subjects. Regurgitation/vomiting yielded the best symptom discrimination in this patient population and was abnormal in 46% of GERD subjects versus only 24% of those with normal pH study results. There was a relatively weak correlation between reflux symptoms and positive pH study results in these 7- to 16-year-old subjects.^21^

There are several limitations in our study. First, normal values for esophageal MII-pH monitoring for infants have not yet been established. Second, we did not measure other extra-esophageal symptoms such as apnea and bradycardia. These other extra-esophageal GERD symptoms might also be associated with GERD in infants. Third, due to ethical considerations of performing invasive tests in healthy infants, we did not enroll a control group to compare the results obtained from the symptomatic infants with GERD who were included in the study. Fourth, our study did not correlate the results of endoscopic and histologic findings with GASQ and MII-pH study results. This information might help us to evaluate the role of the GASQ score for the diagnosis of GERD in infants.

## Summary

To our knowledge, this is the first study to correlate GASQ survey results and esophageal MII-pH monitoring data. GASQ is one of the validated diagnostic questionnaires that may be used to identify infants with GERD who may merit further evaluation. We conclude that an association between esophageal reflux and extra-esophageal symptoms of coughing, gagging, or choking is more likely to be observed in contrast to the lack of an association between GERD and irritability or pain in infants.
